# Epithelial-Mesenchymal Transition Phenotype, Metformin, and Survival for Colorectal Cancer Patients with Diabetes Mellitus II

**DOI:** 10.1155/2017/2520581

**Published:** 2017-06-28

**Authors:** Yaodu Wang, Zhiyang Wu, Likuan Hu

**Affiliations:** ^1^Cancer Center, Shandong University Qilu Hospital, West Wenhua Road 107, Jinan, Shandong Province 250012, China; ^2^Intensive Care Unit, Shandong University Qilu Hospital (Qingdao), Hefei Road 758, Qingdao, Shandong Province 266035, China

## Abstract

**Objectives:**

We aimed to explore the association between metformin treatment and epithelial-mesenchymal transition (EMT) phenotype and further appraise the prognostic values of metformin and EMT markers E-cadherin and vimentin for colorectal cancer (CRC) in clinical practice.

**Methods:**

We collected specimens and evaluated clinicopathological parameters of 102 stage I to III CRC patients with prediagnosed type 2 diabetes mellitus (DM II). Expression of E-cadherin and vimentin in tumors was detected by immunohistochemistry (IHC), and statistical analysis was performed using SPSS 19.0.

**Results:**

In correlation tests, we found a lower tumor cell EMT degree (more E-cadherin (*P* = 0.014) and less vimentin (*P* = 0.011) expression in patients who used metformin, and the expression of E-cadherin and vimentin was associated with serum CA19-9 (*P* = 0.048, *P* = 0.009), tumor invasive depth (T) (*P* < 0.001, *P* = 0.045), and lymph invasion (N) (*P* = 0.013, *P* = 0.001). In Cox multivariate regression analysis, E-cadherin was identified as a prognostic factor for disease-free survival (DFS) (*P* = 0.038) and metformin use (*P* = 0.015*P* = 0.044) and lymph invasion (*P* = 0.016*P* = 0.023) were considered as the prognostic factors for both DFS and overall survival (OS).

**Conclusion:**

Our study suggested that metformin may impede the EMT process and improve survival for stage I–III CRC patients with DM II.

## 1. Introduction

Colorectal cancer (CRC) is the third most common cancer and the fourth leading cause of cancer-related death worldwide [1]. With proper treatments, CRC patients in the early stages have been able to achieve long disease-free survival (DFS). Several studies have indicated that CRC and many other cancer patients with diabetes mellitus (DM) tend to have a worse prognosis than patients without DM [2–5]. Over the last decade, metformin, as a classical oral hypoglycemic agent, has been discovered to possess antineoplastic activities [6, 7] and has been proven effective in survival improvement for CRC patients with type II DM (DM II) [8–12].

Epithelial-mesenchymal transition (EMT) is a process by which epithelial cells lose epithelial characteristics, such as polarity and adhesion, gain migratory properties, and transform into mesenchymal cells [13]. EMT is widely occurring in embryonic development, tissue regeneration, fibrosis, and cancer [14]. EMT has been found to be associated with invasion, metastasis, recurrence, and drug resistance in cancer [15, 16]. Based on a series of antineoplastic evidence from previous reports, the anti-EMT activity of metformin was also tested in a few studies. With metformin exposure, EMT inhibition was observed in breast cancer, melanoma, prostate cancer, lung cancer, and thyroid cancer cells [17–24]. However, all the abovementioned anti-EMT studies were performed using cell lines in vitro, and there is still little analogous research for CRC EMT.

Here, we aim to explore the association between metformin use and EMT degree for CRC patients with DM II in a clinical setting. In our study, clinicopathologic parameters were evaluated and the expression of EMT markers E-cadherin and vimentin in tumor tissue was detected by immunohistochemistry (IHC). Conclusions were based on the outcomes from statistical analysis including correlation tests and survival modeling for variables.

## 2. Materials and Methods

### 2.1. Patients

Patients with DM II, diagnosed pathologically with stage I to III colorectal cancer who underwent radical surgery in Qilu Hospital of Shandong University between 2008 and 2012, were eligible for our study. The clinical parameters of patients were obtained from the electronic medical record (EMR) system and case follow-ups. The exclusion criteria were set as follows: (1) secondary primary malignant tumor; (2) inflammatory bowel disease (IBD) or Peutz-Jeghers syndrome; (3) diagnosed with DM II less than 1 year before tumor diagnosis [25]; (4) administered metformin less than 6 months before tumor diagnosis [2]; (5) pathologic type other than adenocarcinoma; (6) accepted anticancer treatment before surgery; (7) did not reach R0 resection; (8) and incomplete medical records. The primary tumor was evaluated by the tumor-node-metastasis (TNM) staging system (American Joint Committee on Cancer (AJCC), version 7.0), and therapy outcome was assessed by the Response Evaluation Criteria in Solid Tumor (RECIST, version 1.1).

### 2.2. Specimen and IHC

The paraffin-embedded tumor tissues from radical surgery were cut into slices. After dewaxing and dehydrating, slides were subjected to antigen retrieval by citric acid treatment (pH 6.0) and microwave. Slides were then treated with H_2_O_2_ to block endogenous peroxidase activity and subsequently incubated with 5% bovine serum albumin phosphate-buffered saline (BSA-PBS) solution, diluted monoclonal rabbit anti-E-cadherin (Cell Signaling Technology, USA), and antivimentin antibody (Cell Signaling Technology, USA) at 4**°**C overnight. The poly-horseradish peroxidase (Poly-HRP) immunoglobulin G (IgG) detection kit (ZSGB-BIO, China P. R.) and DAB kit (ZSGB-BIO, China P. R.) were used for detection and staining. The DAB-stained slides were counterstained with hematoxylin, dehydrated with alcohol, and cleared with xylene. Slides of normal colon and rhabdomyosarcoma tissue were also tested for the positive controls of E-cadherin and vimentin, respectively. BSA-PBS solution (5%) without antibody was used for negative controls.

The stained slides were scored under a light microscope by 3 pathologists according to the following criteria: (1) intensity of staining: 1 = weak staining, 2 = moderate staining, and 3 = strong staining; (2) percentage of stained cells: 1 = 1–10%, 2 = 11–35%, 3 = 36–65%, and 4 = 66–100%. The total score was calculated by the sum of intensity and percentage scores, and a total score **>** 3 was defined as positive expression. Pathologists participating in scoring were blinded to clinical data.

### 2.3. Statistical Analysis

The primary endpoint of the study was overall survival (OS). Student's *t*-test was used for continuous variable analysis, and Pearson's chi-squared (*χ*^2^) test was used for comparison of categorical variables. Survival data were analyzed using the Kaplan-Meier method and compared by log-rank statistics. Cox regression was used for multivariate survival analysis. Outcomes with a *P* < 0.05 were considered significant. All statistical analyses were performed with SPSS 19.0 software.

### 2.4. Ethics Statement

This study was approved by the Ethics Committee of Qilu Hospital, and all patients provided informed consent.

## 3. Results

### 3.1. Patient Characteristics

**Figure 1:**
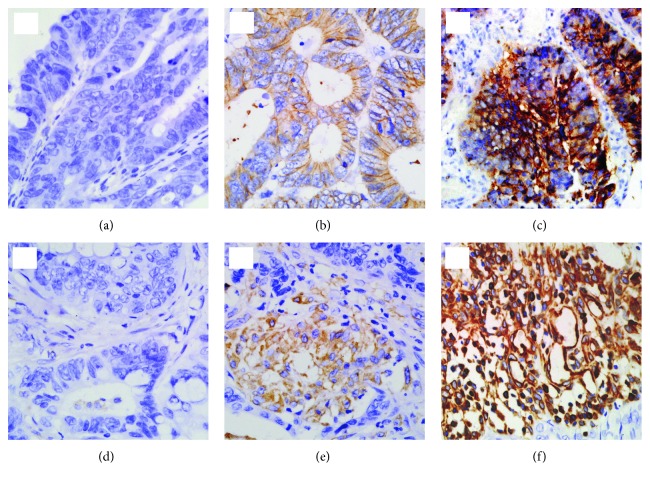
Representative pictures by immunohistochemistry for E-cadherin and vimentin expression in specimen. (Magnification ×400) E-cadherin expression: (a) negative expression, (b) low expression, and (c) high expression. Vimentin expression: (d) negative expression, (e) low expression, and (f) high expression.

A final total of 102 of 115 patients were included in the study (7 patients were excluded because the DM II diagnosis date was less than 6 months before surgery, 2 patients were excluded for receiving presurgery chemotherapy, and 4 patients were lost to follow-up). The median duration of follow-up was 1678 days. Clinicopathologic parameters of the cohort, such as age, gender, smoking and drinking history, family history, tumor location, staging, invasive depth, lymph invasion, and E-cadherin and vimentin expression are shown in Table 1. Of the total patients, 28 (27.45%) were metformin users, 49 (48.04%) were detected as positive for E-cadherin expression, and 55 (53.92%) were detected positive for vimentin expression (representative pictures in Figure 1).

### 3.2. Association between Metformin Use, E-cadherin and Vimentin Expression, and Clinicopathologic Parameters

**Figure 2:**
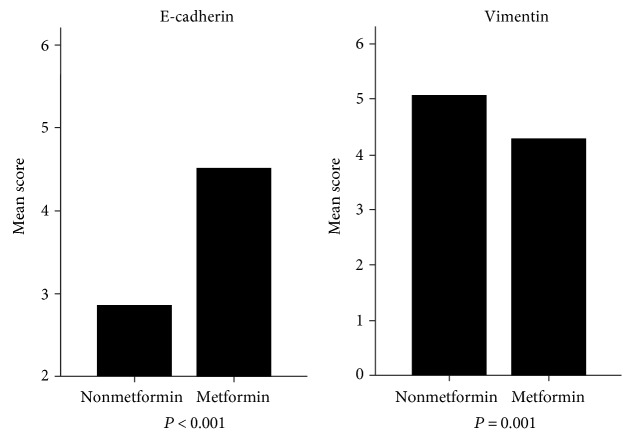
IHC scores of E-cadherin and vimentin in CRC tissues from patients with or without metformin: compared with patients without metformin, patients with metformin expressed higher E-cadherin (*P* < 0.001) and lower vimentin (*P* = 0.001).

As presented in Table 1, insulin injection (*P* = 0.018) and E-cadherin (*P* = 0.014) and vimentin (*P* = 0.011) expression were significantly associated with metformin use. Using the Mann–Whitney *U* test for IHC scores (Figure 2), we further observed trends that patients using metformin expressed higher levels of E-cadherin (*P* < 0.001) and lower levels of vimentin (*P* = 0.001) than those without metformin use. Patients with metformin treatment showed less lymph invasion (*P* = 0.041). Significant relationships were not observed with gender, age, body mass index (BMI), tumor markers, and other variables except family malignant history (*P* = 0.016).

In Table 2, expression of both E-cadherin and vimentin showed significant associations with tumor invasive depth (T) (*P* < 0.001, *P* = 0.045) and lymph invasion (N) (*P* = 0.013, *P* = 0.001). As presurgery tumor markers, E-cadherin was correlated with both serum CEA (*P* = 0.007) and CA19-9 (*P* = 0.048) and vimentin was correlated with serum CA19-9 (*P* = 0.009). No significant associations were observed for tumor site, histologic grade, sulfonylurea use, or other parameters.

### 3.3. Survival Analysis

Recurrence was diagnosed in 46 (45.01%) patients and death occurred in 37 (36.27%) patients by the end of follow-up.

Univariate survival analysis using the Kaplan-Meier method (Table 3) showed that normal presurgery serum CA19-9 values (*P* = 0.006, *P* = 0.005), metformin use (*P* = 0.003, *P* = 0.004), shallow tumor invasive depth (T1–3) (*P* = 0.004, *P* = 0.010), less lymph invasion (N) (*P* < 0.001, *P* < 0.001), positive E-cadherin expression (*P* = 0.001, *P* = 0.003), and negative vimentin expression (*P* = 0.019, *P* = 0.012) contributed to both longer DFS and OS. Alcohol drinking, smoking, family history, serum CEA level presurgery, sulfonylurea or insulin use, tumor histologic grade, and adjuvant chemotherapy were not associated with survival.

The prognostic values of variables were further tested by multivariate Cox regression (Table 4). Metformin use (*P* = 0.015, *P* = 0.044) and lymph invasion (*P* = 0.016, *P* = 0.023) were confirmed as significant independent prognostic factors for both DFS and OS. E-cadherin expression (*P* = 0.038) was considered to be a prognostic factor only for DFS. Alcohol drinking (*P* = 0.035) was determined to be a risk factor for DFS, whereas tumor invasive depth (*P* = 0.066, *P* = 0.108) and vimentin expression (*P* = 0.369, *P* = 0.900), which were significant in Kaplan-Meier analysis, were not identified as prognostic factors in the multivariate model.

## 4. Discussion

Our study explored the association between metformin use and EMT marker (E-cadherin and vimentin) expression in 102 CRC patients with DM II and further estimated the prognostic values of metformin, EMT markers, and other clinicopathologic parameters.

As common molecular markers for EMT studies, E-cadherin performs cell to cell adhesive functions for the epithelium and is conventionally used to measure cell epithelization [26]; vimentin is a major cytoskeletal component for mesenchymal cells and is routinely applied in identifying mesenchyme [27]. Correlation analysis in our study revealed the expression discrepancy of E-cadherin and vimentin between the metformin group and the nonmetformin group: patients in the metformin group showed higher E-cadherin and lower vimentin expression in tumors, indicating lower proportions of EMT cells and lower EMT degree of malignant tissues. The results suggested that metformin use may impede the EMT process of CRC.

Among numerous studies on metformin's antiplastic mechanisms, EMT inhibition was first reported in MDA-MB-231 and transforming growth factor beta- (TGF-*β*-) induced MCF-7 breast cancer cells [17, 18]. This effect was discovered with the regulation of the cancer stem cell phenotype and has been further verified in other cell lines by previous studies [19–24]. To the best of our knowledge, this is the first study to reveal the anti-EMT potential of metformin in CRC. Although analogous results had not been directly reported in previous laboratory studies, the following clues were adequate to support our conclusion.

According to existing studies, the main mechanism of metformin action in DM II treatment is AMP-activated protein kinase (AMPK) activation [28]. By activating AMPK and downstream messengers, metformin suppresses gluconeogenesis, downregulates blood sugar and insulin levels, and moderates the metabolism of glucose and fat [29]. Similarly, in the anticancer studies, researchers have found that metformin can impede tumor growth by activating the AMPK pathway [30, 31]. For human colorectal cell lines HT-29 and drug-resistant cell line LoVo, metformin was observed to activate AMPK and inhibit cell growth [32, 33]. Additionally, AMPK pathway activation was considered as an alternative approach to inhibit EMT [34], and metformin has also been proven to perform such inhibition through AMPK activation for melanoma and breast cancer cells [19, 20].

Researchers have also confirmed that several other signaling pathways such as TGF-*β*, JAK-STAT, PI3K/Akt/mTOR, and Wnt/*β*-catenin [35–38] are involved in EMT occurrence and development, and metformin was observed to act on these pathways [18, 39–41]. Particularly, Zhao et al. discovered that metformin could inhibit IL-6-induced lung cancer EMT by blocking STAT3 phosphorylation [22], Han et al. found the metformin could suppress EMT of thyroid cells through inhibiting mTOR [24], and Banerjee et al. demonstrated that metformin can delay EMT development by interfering with Wnt signaling [41]. Although analogous results had not been reported for CRC, relevant implications and clues were adequately offered from abovementioned studies.

In addition to metformin use, our study revealed that normal presurgery serum CEA and CA19-9 levels, tumor invasion beneath the visceral peritoneum (T1–3), and negative lymph invasion (N0) were associated with positive E-cadherin expression, and high presurgery serum CEA levels, insulin injection, invasion through visceral peritoneum (T4), and positive lymph invasion (N1-2) were associated with positive vimentin expression. A few studies also investigated the relationship between CA19-9 and EMT. For CEA and EMT, in one study, we found that soluble CEA molecules can not only enhance colorectal cell growth but also bind to the TGF-*β* receptor, which is a common trigger for EMT and inhibits TGF-*β* signaling [42]. This case was in contrary to our results, but we propose that the inhibition effect was not sufficient to block the mainstream of EMT because there was no further supporting evidence. However, in clinical practice for CRC, abnormally high CEA and CA19-9 levels in serum are more common in patients with advanced disease [43, 44]. According to previous studies, E-cadherin and vimentin expression was associated with tumor stage, which was in accordance with our results. Yagasaki et al. discovered that E-cadherin and vimentin are associated with axillary metastases in breast cancer [45], and Liu et al. discovered that low E-cadherin expression is correlated with TNM stage for basal-like breast cancer [46]. Moreover, E-cadherin and vimentin were also determined to be associated with lymph invasion and TNM stage for lung squamous carcinoma and oral squamous carcinoma [47, 48]. It should be noted that 3 of the studies mentioned above [45–47] also found the correlation between E-cadherin and vimentin expression and tumor histologic grade; however, such correlation was not significant in our study.

Although patients with normal serum CA19-9 showed significantly longer DFS (*P* = 0.006) and OS (*P* = 0.005) in the Kaplan-Meier analysis, in Cox regression, neither CEA nor CA19-9 was considered as prognostic factors (Table 4), which did not accord with the common views of biomarkers' clinical values. We attribute the results to the following three aspects: (1) limited sample size; (2) low mortality (36.37%) of local stage (I–III) colorectal cancer in the follow-up period (median: 1678 days); (3) Kaplan-Meier curve and Cox regression were used only for survival data classified by categorical factors or cut-off values. As continuous variables, CEA and CA19-9 were redefined to categorical factors by cut-off values; thus, the original characteristics were inevitably ignored.

The prognostic values of E-cadherin and vimentin have been widely reported in previous studies. E-cadherin and vimentin were found to be prognostic factors for both DFS and OS for lung cancer [47, 49] and oral squamous cancer [50]. E-cadherin has also been suggested as a positive indicator for OS for cervical squamous carcinoma [51] and CRC [52]. In our Kaplan-Meier survival analysis, E-cadherin and vimentin expression was demonstrated to be significant for both DFS and OS. However, in further multivariate Cox regression, only E-cadherin was identified as a prognostic factor for DFS. The contradictory results from Cox regression might be attributed to our limited sample size and inclusion criteria, as CRC patients without DM II were excluded from our study. However, the positive result of Kaplan-Meier analysis could still indicate the prognostic potential of E-cadherin and vimentin.

Patients with metformin not only revealed less EMT in tumors but also showed longer DFS and OS than patients who did not choose metformin for DM II treatment. However, it remains controversial whether metformin could bring survival benefits for cancer patients with DM II. For CRC, our results were supported by several studies [8–12] but do not agree with the results of 2 recent studies [53, 54]. On the other hand, 6 of 7 studies were retrospective and the remaining study is a meta-analysis [12]. Therefore, the conflicting conclusions need to be verified by a multicenter prospective study with a large cohort.

In summary, combining the result of correlation analysis and survival analysis described above, we suggest that EMT is correlated with CRC progression and can be impeded by metformin. Metformin use and positive E-cadherin expression indicate better prognosis for CRC patients with DM II. Additionally, it is reasonable to believe that the antineoplastic activity of metformin as partially implemented by EMT interference. To further understand the anti-EMT effect of metformin, more inner molecular mechanisms need to be explored in future studies.

## Figures and Tables

**Table 1:** Clinicopathologic characteristics of 102 patients.

Variable	Category	Metformin group	Nonmetformin group	*P*
Age		63.57, mean	63.74, mean	0.596

Gender	Female	10	25	0.855
	Male	18	49	

Drinking history	Yes	8	12	0.161
	No	20	62	

Smoking history	Yes	7	23	0.547
	No	21	51	

Family history	Yes	5	2	0.016^∗^
	No	23	72	

BMI		25.46, mean	24.73, mean	0.889

CEA		5.68, mean	4.11, mean	0.228

CA19-9		9.7, mean	11.97, mean	0.172

Sulfonylurea	Yes	13	34	0.965
	No	15	40	

Insulin	Yes	4	30	0.018^∗^
	No	24	44	

Site	Right	7	12	0.787
	Transverse	1	4	
	Left	1	5	
	Sigmoid	6	13	
	Rectum	13	40	

Histologic grade	I-II	22	63	0.427
	III	6	11	

T	T1–3	18	34	0.098
	T4	10	40	

N	N0	21	39	0.041^∗^
	N1-2	7	35	

E-cadherin	Positive	19	30	0.014^∗^
	Negative	9	44	

Vimentin	Positive	8	42	0.011^∗^
	Negative	20	32	

^∗^ *P* < 0.05.

**Table 2:** Associations between expression of E-cadherin, vimentin, and clinicopathologic characteristics.

Variable	Category	E-cadherin		*P*	Vimentin		*P*
		Positive	Negative		Positive	Negative	
Age		64.24, mean	63.19, mean	0.639	64.62, mean	62.69, mean	0.501

Gender	Female	15	20	0.449	18	17	0.715
	Male	34	33		37	30	

Drinking history	Yes	9	11	0.762	7	13	0.058
	No	40	42		48	34	

Smoking history	Yes	16	14	0.490	16	14	0.939
	No	33	39		39	33	

Family history	Yes	4	3	0.708	3	4	0.543
	No	45	50		52	43	

BMI		25.04, mean	24.83, mean	0.876	24.68, mean	25.2, mean	0.888

CEA		8.24, mean	14.23, mean	0.007^∗^	13.76, mean	8.75, mean	0.105

CA19-9		23.46, mean	36.58, mean	0.048^∗^	42.27, mean	17.31, mean	0.009^∗^

Sulfonylurea	Yes	27	20	0.079	24	23	0.592
	No	22	33		31	24	

Insulin	Yes	13	21	0.161	24	11	0.032^∗^
	No	36	32		31	36	

Site	Right	9	10	0.934	9	10	0.770
	Transverse	2	3		2	3	
	Left	2	4		4	2	
	Sigmoid	10	9		9	10	
	Rectum	26	27		31	22	

Histologic grade	I-II	40	45	0.658	43	42	0.131
	III	9	8		12	5	

T	T1–3	35	17	<0.001^∗^	23	29	0.045^∗^
	T4	14	36		32	18	

N	N0	35	25	0.013^∗^	24	36	0.001^∗^
	N1-2	14	28		31	11	

^∗^ *P* < 0.05.

**Table 3:** Kaplan-Meier analysis for disease-free survival (DFS) and overall survival (OS).

Variable	Category	Recurrence		*P*	Death		*P*
		Yes	No		Yes	No	
Age (year)	≤50	7	8	0.942	6	9	0.758
	>50	39	48		31	56	

Gender	Female	14	21	0.775	11	24	0.562
	Male	32	35		26	41	

Drinking history	Yes	5	15	0.080	4	16	0.098
	No	41	41		33	49	

Smoking history	Yes	12	18	0.324	9	21	0.152
	No	34	38		28	44	

Family history	Yes	3	4	0.786	3	4	0.973
	No	43	52		34	61	

BMI (kg/m^2)	≤25	22	31	0.633	20	33	0.598
	>25	24	25		17	32	

CEA (ng/ml)	≤5	23	35	0.279	18	39	0.650
	>5	23	21		19	26	

CA19-9 (U/ml)	≤35	32	51	0.006^∗^	25	59	0.005^∗^
	>35	14	5		12	6	

Sulfonylurea	Yes	19	28	0.268	15	32	0.371
	No	27	28		22	33	

Insulin	Yes	18	17	0.293	14	21	0.486
	No	28	39		23	44	

Metformin	Yes	7	21	0.003^∗^	6	22	0.004^∗^
	No	39	35		31	43	

Site	Right	7	12	0.460	6	13	0.876
	Transverse	3	2		2	3	
	Left	1	5		1	5	
	Sigmoid	8	11		7	12	
	Rectum	27	26		21	32	

Adjuvant chemotherapy	Yes	25	24	0.243	20	29	0.321
	No	21	32		17	36	

Histologic grade	I-II	39	46	0.992	31	54	0.866
	III	7	10		6	11	

T	T1–3	17	35	0.004^∗^	13	39	0.010^∗^
	T4	29	21		24	26	

N	N0	17	43	<0.001^∗^	14	46	<0.001^∗^
	N1-2	29	13		23	19	

E-cadherin	Positive	15	34	0.001^∗^	12	37	0.003^∗^
	Negative	31	22		25	28	

Vimentin	Positive	31	24	0.019^∗^	26	29	0.012^∗^
	Negative	15	32		11	36	

^∗^ *P* < 0.05.

**Table 4:** Multivariate Cox analysis of prognostic factors for DFS and OS.

Variable	Category	DFS			OS		
		HR (95% CI)	*χ* ^2^	*P*	HR (95% CI)	*χ* ^2^	*P*
Age (year)	≤50	0.898 (0.365–2.213)	0.054	0.816	0.789 (0.264–2.359)	0.180	0.671
	>50						

Gender	Female	0.547 (0.250–1.198)	2.274	0.132	0.418 (0.168–1.040)	3.516	0.061
	Male						

Drinking history	Yes	3.451 (1.092–10.913)	4.448	0.035^∗^	2.578 (0.737–9.019)	2.198	0.138
	No						

Smoking history	Yes	1.325 (0.603–2.912)	0.491	0.484	2.358 (0.875–6.356)	2.873	0.090
	No						

Family history	Yes	0.659 (0.162–2.671)	0.342	0.559	0.523 (0.106–2.582)	0.634	0.426
	No						

BMI (kg/m^2)	≤25	0.711 (0.336–1.508)	0.790	0.673	1.457 (0.618–3.433)	0.740	0.390
	>25						

CEA (ng/ml)	≤5	0.857 (0.418–1.756)	1.179	0.357	0.857 (0.369–1.994)	0.128	0.721
	>5						

CA19-9 (U/ml)	≤35	0.694 (0.319–1.509)	0.850	0.374	0.629 (0.260–1.520)	1.060	0.303
	>35						

Sulfonylurea	Yes	1.629 (0.831–3.192)	2.019	0.155	1.672 (0.758–3.690)	1.623	0.203
	No						

Insulin	Yes	1.127 (0.562–2.261)	0.114	0.736	1.189 (0.545–2.596)	0.189	0.664
	No						

Metformin	Yes	3.450 (1.273–9.351)	5.924	0.015^∗^	3.484 (1.036–11.723)	4.066	0.044^∗^
	No						

Site	Right	0.871 (0.686–1.106)	1.282	0.258	0.978 (0.744–1.285)	0.026	0.871
	Transverse						
	Left						
	Sigmoid						
	Rectum						

Adjuvant chemotherapy	Yes	0.635 (0.315–1.280)	1.614	0.204	0.713 (0.312–1.627)	0.647	0.421
	No						

Histologic grade	I-II	1.006 (0.357–2.839)	0.001	0.991	1.038 (0.332–3.241)	0.004	0.949
	III						

T	T1–3	0.513 (0.252–1.046)	3.368	0.066	0.513 (0.227–1.159)	2.577	0.108
	T4						

N	N0	0.382 (0.175–0.834)	5.829	0.016^∗^	0.362 (0.150–0.872)	5.132	0.023^∗^
	N1-2						

E-cadherin	Positive	2.252 (1.046–4.849)	4.303	0.038^∗^	1.923 (0.824–4.488)	2.284	0.131
	Negative						

Vimentin	Positive	1.409 (0.667–2.975)	0.807	0.369	1.056 (0.455–2.451)	0.016	0.900
	Negative						

^∗^ *P* < 0.05.

## Abbreviations

CRC: Colorectal cancer
EMT: Epithelial-mesenchymal transition
CA19-9: Carbohydrate antigen 19-9
CEA: Carcinoembryonic antigen
AMPK: Adenosine monophosphate-activated protein kinase
TGF-*β:*: Transforming growth factor beta
JAK: Janus kinase
STAT: Signal transducer and activator of transcription
mTOR: Mammalian target of rapamycin
DFS: Disease-free survival
OS: Overall survival.
